# Cardiac output estimation using pulmonary mechanics in mechanically ventilated patients

**DOI:** 10.1186/1475-925X-9-80

**Published:** 2010-11-25

**Authors:** Ashwath Sundaresan, J Geoffrey Chase, Christopher E Hann, Geoffrey M Shaw

**Affiliations:** 1 Department of Mechanical Engineering, College of Engineering, University of Canterbury, Private Bag 8140, Christchurch, New Zealand; 2 Department of Intensive Care, Christchurch Hospital, Private Bag 4710, Christchurch, New Zealand

## Abstract

The application of positive end expiratory pressure (PEEP) in mechanically ventilated (MV) patients with acute respiratory distress syndrome (ARDS) decreases cardiac output (CO). Accurate measurement of CO is highly invasive and is not ideal for all MV critically ill patients. However, the link between the PEEP used in MV, and CO provides an opportunity to assess CO via MV therapy and other existing measurements, creating a CO measure without further invasiveness.

This paper examines combining models of diffusion resistance and lung mechanics, to help predict CO changes due to PEEP. The CO estimator uses an initial measurement of pulmonary shunt, and estimations of shunt changes due to PEEP to predict CO at different levels of PEEP. Inputs to the cardiac model are the PV loops from the ventilator, as well as the oxygen saturation values using known respiratory inspired oxygen content. The outputs are estimates of pulmonary shunt and CO changes due to changes in applied PEEP. Data from two published studies are used to assess and initially validate this model.

The model shows the effect on oxygenation due to decreased CO and decreased shunt, resulting from increased PEEP. It concludes that there is a trade off on oxygenation parameters. More clinically importantly, the model also examines how the rate of CO drop with increased PEEP can be used as a method to determine optimal PEEP, which may be used to optimise MV therapy with respect to the gas exchange achieved, as well as accounting for the impact on the cardiovascular system and its management.

## Introduction

Patients diagnosed with acute respiratory distress syndrome (ARDS) often experience pulmonary shunt or gas exchange abnormalities due to lack of recruited alveoli. Shunt also occurs when there is an increased thoracic pressure that restricts blood flow, thus reducing the gas exchange. The primary objective of mechanical ventilation (MV) is to improve the condition of the patient by increasing alveoli recruitment and thus also improving or optimising gas exchange.

A common MV parameter used to optimise recruitment is the positive end expiratory pressure (PEEP). PEEP is used to prevent alveoli derecruitment at the end of the expiration cycle [1] and to maintain a level of oxygenation. However, clinicians often debate the optimal level of PEEP required [2-4]. In particular, the application of PEEP poses some unique problems that need to be addressed.

First, the ranges of allowable PEEP for ventilation vary significantly between patients [5]. If PEEP is set too high, healthy, non-ARDS alveoli may over inflate, which can cause more harm to the patient [6]. However, if PEEP is too low, then cyclic derecruitment can occur during the breathing cycle, which can aggravate and further damage the alveoli [7]. This balance results from the heterogeneous nature of the ARDS lung with a mix of healthy and ARDS affected alveoli at all levels [8].

Second, the application of PEEP decreases cardiac output (CO) [9,10]. The decrease in CO from high PEEP is due to the reduction in stroke volume (SV). As PEEP increases, the intrathoracic pressure increases, which restricts the venous flow into the thorax and thus lowers CO. Any decrease in CO lowers oxygen consumption, as determined by Fick's law, and describes the inability of the heart to pump enough blood to meet the metabolic requirements of the body. Reduced CO can also have an impact on circulation management and therapeutics used. Hence, PEEP may also have a negative spill over into cardiovascular therapy.

Current methods to measure CO, such as thermodilution and pulse pressure methods, are clinically invasive, requiring the use of catheters [11,12]. These catheters carry some added risks of infection [13], and are thus used only in a subset of critically ill patients receiving MV. For critically ill patients, the benefits from CO measurements may not offset the dangers associated with invasive tools. In addition, these methods take time and effort, and are thus not performed regularly and cannot be monitored in clinical real time. Hence, strong motivations exist to develop non-invasive, real-time tools to measure CO.

Thus, MV patients treated for ARDS may not have CO measurements readily available. However, the changes in CO due to changes in applied PEEP are important as it may also help indicate an optimal level of PEEP. In particular, if a change in PEEP causes a large drop in CO, it may more than offset its benefit on recruitment. Alternatively, if the drop in CO is minimal, then the benefits of PEEP induced recruitment may offset the detrimental effects of an insignificant drop in CO. Hence, the ability to model changes in CO due to PEEP may also prove beneficial for optimizing the setting of PEEP, which is itself controversial [4].

Simple mathematical models and parameter identification methods can provide the framework to quantify unknown physiological values using known clinical information. Mathematical models can also allow clinicians to assess the impact of various therapies without having to implement them. Finally, such model-based approaches can create a clinical, physiological picture of the patient to clearly illustrate tradeoffs between condition and treatment choices. Thus, there is potential to mitigate harmful effects that can occur with non-optimal clinical decisions.

## Model Based Methods

This paper examines two separate mathematical models and aims to combine the effects of both to estimate changes in CO due to changes in PEEP. The first model, developed by Andreassen et al [14] looks at estimations of pulmonary shunt and oxygen diffusion resistance by measuring variations in the fraction of inspired oxygen (FIO_2_) and arterial oxygen saturation (S_a_O_2_). The diffusion model takes inputs of CO, F_I_O_2 _and other ventilation data, to estimate pulmonary shunt and diffusion resistance as outputs.

The second model was developed by Sundaresan et al [15,16] and evaluates the effect of PEEP on alveolar recruitment. In particular, the model quantifies the level of alveolar recruitment as a function of PEEP by evaluating the threshold opening and closing pressures required to recruit and derecruit alveoli. It obtains these values based on clinically measured respiratory PV loops from MV patients.

### Diffusion Model

Current methods of describing gas exchange abnormalities, such as arterial oxygen saturation, alveolar arterial oxygen pressure gradient or venous admixture [17,18] are insufficient. In particular, these parameters lump the effects of oxygen diffusion and true pulmonary shunt into a single parameter. This single parameter is typically inadequate as it is difficult to interpret which mechanism causes the gas exchange abnormality.

Pulmonary shunt occurs when the alveoli in the lung are perfused with blood, as desired, but not adequately ventilated. In patients with ARDS, alveoli collapse occurs as a result of fluid build up, which results in the ARDS lung not being ventilated. Thus, as ARDS severity increases, pulmonary shunt also increases. Even if the alveoli is recruited or not collapsed, if gas exchange does not occur it is considered to be part of the shunt volume. Similar abnormality or failure of gas exchange can occur when CO and minute ventilation are mismatched [19].

In patients who are mechanically ventilated, the application of PEEP can increase alveolar recruitment. As more alveoli are recruited, there is an increase in alveolar ventilation and as a result, pulmonary shunt decreases. Using standard data, such as S_a_O_2 _and arterial oxygen pressure (P_a_O_2_), curves of S_a_O_2 _can be generated as a function of F_I_O_2_. These curves can then be used to estimate the pulmonary shunt and diffusion resistance [20,21].

**Figure 1:**
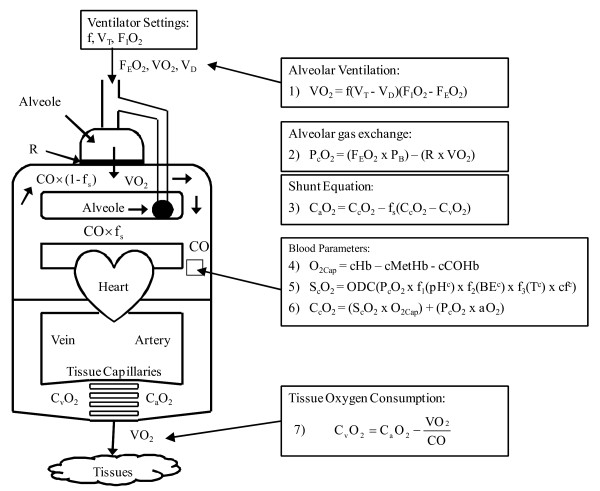
**Diffusion Model reproduced from Rees et al **[22].

The model developed by Andreassen et al [14] uses a compartmental oxygen status model (OSM) as shown in Figure 1.

The net oxygen consumption by the alveoli (VO_2_) is calculated by using known values F_I_O_2_, fraction of expired oxygen (F_E_O_2_), respiratory frequency (f) and tidal volume (V_T_), and where dead space (V_D_) is the amount of air in the lungs that does not reach the alveoli, and thus is does not contribute to any gas exchange.

(1) $$\text{V}{\text{O}}_{2}=\text{f}({\text{V}}_{\text{T}}-{\text{V}}_{\text{D}})({\text{F}}_{\text{I}}{\text{O}}_{2}-{\text{F}}_{\text{E}}{\text{O}}_{2})$$

Once the inspired gas reaches the alveolar compartments, oxygen begins the diffusion process across the alveolar membrane to the capillaries. The partial pressure of oxygen in the capillary (P_c_O_2_) is a function of the partial pressure in the alveoli, which is a function of atmospheric pressure (P_B_), minus the drop in partial pressure due to diffusion resistance (R).

(2) $${\text{P}}_{\text{c}}{\text{O}}_{2}=({\text{F}}_{\text{E}}{\text{O}}_{2}\times {\text{P}}_{\text{B}})-(\text{R}\times \text{V}{\text{O}}_{2})$$

After the oxygen has diffused through the alveolar wall, oxygen from the capillaries with high concentration (C_c_O_2_) mixes with venous blood, which has low concentration (C_v_O_2_). Depending on the level of pulmonary shunt (f_s_), the arterial oxygen concentration can be evaluated (C_a_O_2_).

(3) $${\text{C}}_{\text{a}}{\text{O}}_{2}={\text{C}}_{\text{c}}{\text{O}}_{2}-{\text{f}}_{\text{s}}({\text{C}}_{\text{c}}{\text{O}}_{2}-{\text{C}}_{\text{v}}{\text{O}}_{2})$$

The oxygen carrying capacity of haemoglobin (O_2Cap_) can be defined as a function of blood parameters such as haemoglobin (cHb), methaemoglobin (cMetHb), carboxyhaemoglobin (cCOHb).

(4) $${\text{O}}_{2}{}_{\text{C}\text{a}\text{p}}=\text{c}\text{H}\text{b}-\text{c}\text{M}\text{e}\text{t}\text{H}\text{b}-\text{c}\text{C}\text{O}\text{H}\text{b}$$

The oxygen saturation curve (S_c_O_2_) can then be calculated based on the oxygen dissociation curve (ODC).

(5) ScO2=ODC(PcO2×f1(pHc)×f2(BEc)×f3(Tc)×cfc)

The ODC is a function of the capillary pH (pH^c^), base excess (BE^c^) and the temperature of the blood (T^c^). Any other variables which influence the ODC are lumped into a correction factor (cf^c^). The ODC is then calculated by multiplying these parameters with the P_c_O_2 _and individual correction factors (f_1_, f_2 _and f_3_). The values of f_1_, f_2 _and f_3 _are obtained from [22].

Finally, the capillary oxygen concentration (CcO_2_) can then be defined:

(6) CcO2=(ScO2×O2Cap)+(PcO×2αO2)

where αO_2 _is the solubility coefficient of oxygen in blood.

The net difference between the arterial concentration and the drop in oxygen consumption by the tissues then gives the venous oxygen concentration (C_v_O_2_).

(7) $${\text{C}}_{\text{v}}{\text{O}}_{2}={\text{C}}_{\text{a}}{\text{O}}_{2}-\frac{\text{V}{\text{O}}_{2}}{\text{C}\text{O}}$$

Table 1 shows the different parameters that are measured, estimated and calculated in the diffusion resistance model. First, the model requires a gas exchange analyser to measure the F_I_O_2 _and F_E_O_2 _values along with a pulse oximeter to measure the S_a_O_2_. Respiratory frequency and tidal volume are measured with a ventilator, while the haemoglobin concentrations are measured by taking a blood sample.

**Table 1:** Measured, estimate and calculated parameters in the diffusion resistance model

Directly Measured Parameters	Estimated Parameters	Calculated Parameters
*Respiratory Frequency *(f)	*Dead Space *(V_d_)	*Shunt *(f_s_)

*Inspired Oxygen Content *(F_i_O_2_)		*Diffusion Resistance *(R)

*Expired Oxygen Content *(F_e_O_2_)		

*Tidal Volume *(V_t_)		

*Atmospheric Pressure *(P_B_)		

*Pulse or Arterial Oxygen Saturation *(S_a_O_2_)		

*Cardiac Output *(CO)		

*Haemoglobin *(cHb)		

*Methaemoglobin *(cMetHb)		

*Carboxyhaemoglobin *(cCOHb)		

In the model developed by Andreassen et al [14], CO was estimated, but it can also be measured using thermodilution techniques. Using all these measurements, and an estimate of dead space, it is then possible to calculate shunt and diffusion resistance by solving Equations (1) - (7).

### Lung Mechanics

The lung mechanics model developed by Sundaresan et al [15,16] considers the lung as a collection of lung units, each representing a set of distal airways and alveoli. The model assumes that any volume change is predominantly due to alveoli recruitment and derecruitment in the ARDS lung.

The recruitability of the lung units is determined by the threshold opening pressure (TOP) and threshold closing pressure (TCP). The TOP is the critical pressure required to recruit an alveolus, while the TCP is the pressure where alveoli collapse, and both are assumed to take on a normal distribution [1]. The TCP and TOP distributions fitted to the data capture the continuous recruitment and derecruitment across a wide range of pressures and are described by a mean and standard deviation. In practice, TOP and TCP distributions are experimentally obtained, but the model allows these distributions to be estimated.

**Figure 2:**
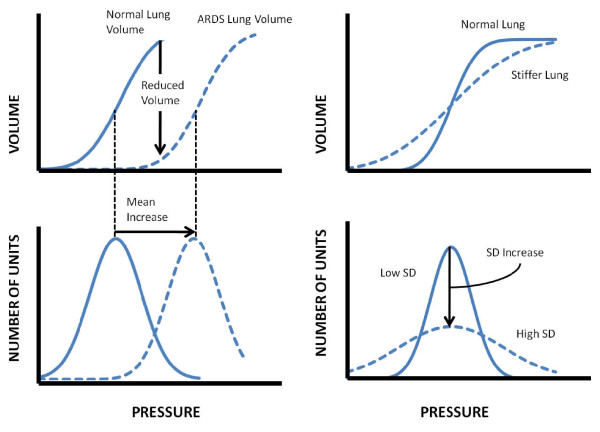
**Physiological relevance of the mean and standard deviation**.

The model uses the standard deviation and mean of these distributions to track changes in patient conditions and responsiveness to MV therapy. As a patient's disease state evolves, the shapes of the distributions are also modified to reflect physiological changes occurring in the lungs, as illustrated in Figure 2. The model is patient-specific, as for a given PEEP, each patient may exhibit a different TOP and TCP mean and SD. The effect of PEEP on recruitment is then measured by evaluating the differences in the TOP and TCP as a function of PEEP, which is essentially the recruited volume response of the ARDS lung to changes in PEEP as assessed by the model.

**Figure 3:**
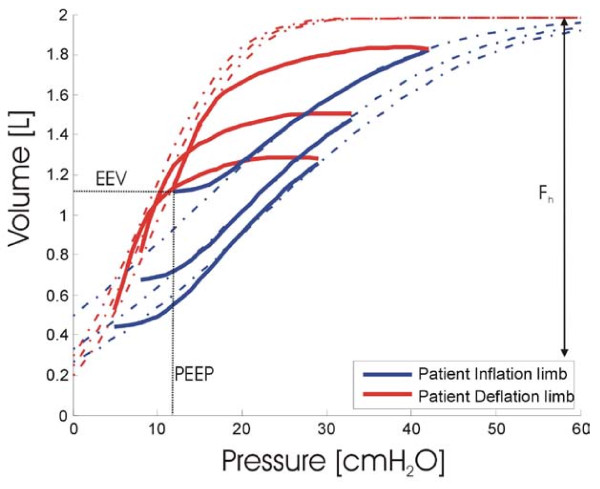
**Model fitting using measured PV loops**. End expiratory volume (EEV) is measured and then all PV loops fitted to maximum theoretical lung volume (F_h_). Data from Bersten et al [23] as used in Sundaresan et al [16].

The model can evaluate the theoretical maximum lung capacity (F_h_) for a given patient if two or more PV loops are known. Fitting a cumulative normal distribution based on the TCP and TOP parameters, the model then predicts the volume that the lung will achieve given a known pressure, as illustrated in Figure 3.

## Model Development and Method

### Diffusion Model Reproduction and Impact of Co

In Andreassen et al [14], the diffusion model was used to simulate responses to variations in the model parameters. In particular, the study looked at how shunt and oxygen diffusion resistance vary under different F_I_O_2_. The simulations used the input parameters shown in Table 2.

**Table 2:** Diffusion model parameters from Andreassen et al 14

Respiratory frequency	f	14 min^-1^
Tidal volume	V_t_	0.5 L

Dead space volume	V_d_	0.15 L

Cardiac output	CO	5 L/min

Oxygen consumption	VO_2_	11.5 mmol/min

Haemoglobin	cHb	9 mmol/L

Methaemoglobin	cMetHb	0 mmol/L

Carboxyhaemoglobin	cCOHb	0 mmol/L

Oxygen solubility coefficient	αO_2_	0.0102 mmol/(L kPa)

Arterial pH status	pH^a^	7.4

Capillary pH status	pH^c^	7.4

Arterial base excess	BE^a^	0 mmol/L

Capillary base excess	BE^c^	1 mmol/L

Arterial blood temperature	T^a^	37 C

Capillary blood temperature	T^c^	37 C

Barometric pressure	P_B_	101.3 kPa

**Figure 4:**
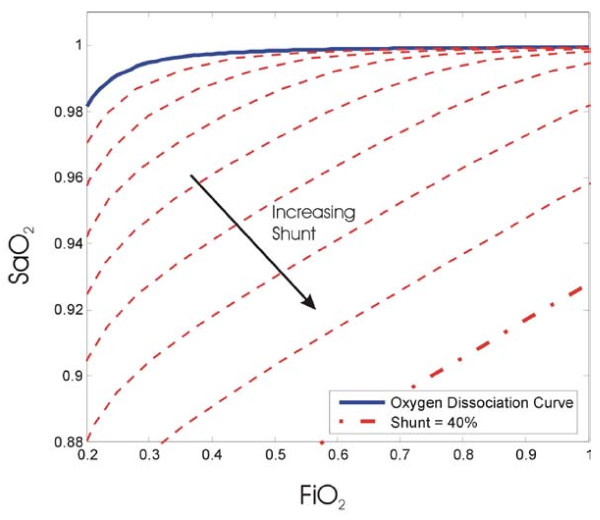
**Oxygen saturation curves changing as a function of F_i_O_2 _and various levels of shunt**.

Figure 4 shows the S_a_O_2 _varying as a function of F_I_O_2 _depending on the level of shunt with a diffusion resistance of zero. When no shunt exists, the S_a_O_2 _curve is identical to the oxygen dissociation curve. However, as shunt increases, then for a given F_I_O_2_, the amount of oxygen saturation decreases.

**Figure 5:**
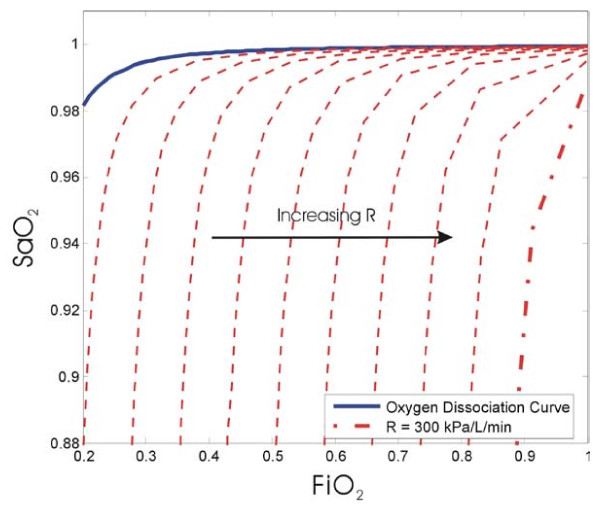
**Oxygen saturation curves at various levels of diffusion resistance and shunt = 0**.

Figure 5 shows the effect of varying the diffusion resistance instead of shunt. Similar to shunt, an increase in oxygen resistance also causes incomplete oxygenation. It is assumed that shunt is zero for all the curves in this figure.

Theoretically, if two different F_I_O_2 _measurements were taken and the corresponding S_a_O_2 _values measured and plotted, it is possible to evaluate the shunt and diffusion resistance (R) by plotting the best fit S_a_O_2 _curve. However, in Figure 4 and 5, the cardiac output is held at a constant value of 5 L/min. Unless measured, the value of CO is assumed to be 5 L/min [14,23]. However, in patients with ARDS where PEEP is titrated based on clinical choice, the assumption of using a constant CO is not valid. In addition, as a result of MV therapy and other aspects of their condition, CO is much more variable within a typical range of 2-7 L/min [24-26].

**Figure 6:**
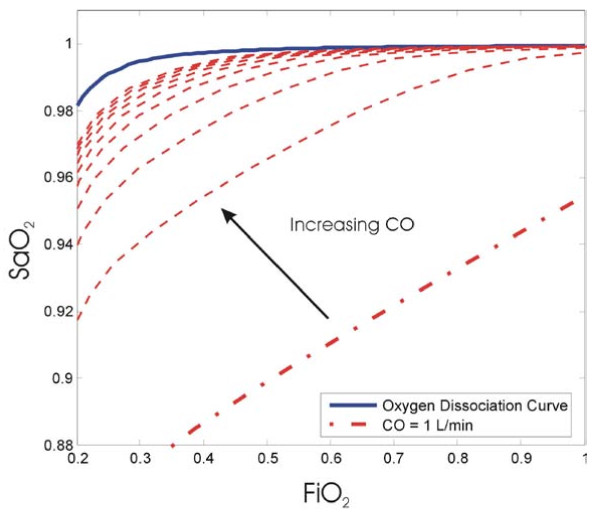
**Oxygen saturation curve varying as a function of cardiac output**.

While such changes in CO were not modelled by Andreassen et al, they are easily incorporated into this model. Figure 6 shows the effect of varying cardiac output given a value of shunt of 10% and a diffusion resistance of 0 kPa/L/min. As shown in Figure 6 an increase in cardiac output causes a more complete oxygenation of the blood. Overall, it adds a third unknown variable (CO) to the shunt and diffusion resistance noted previously.

In Figure4-F the lines of constant shunt, diffusion resistance and cardiac output are analogous to principles in thermodynamics, such as isotherms (lines of constant temperature), isobars (lines of constant pressure) and isochors (lines of constant volume). In particular, if two states are known, then it is possible to determine the third state of a gas as all variables are interrelated. Similarly, the lines of constant shunt, diffusion resistance and cardiac output are all interrelated.

### Estimating Shunt Changes Due to Peep - Linking the Two Models

Because the current diffusion models use CO as an input, it is possible to estimate the shunt and diffusion resistance if a few measurements such S_a_O_2 _and F_I_O_2 _are taken. However, as PEEP increases, the level of alveoli recruitment also increases at the cost of decreased CO. Thus, unless the patient has a continuous measurement of CO, then an alternative method must be developed to generate the S_a_O_2 _curves and predict shunt.

The hypothesis that this research suggests is that any increase in PEEP directly causes a decrease in shunt. In ARDS affected lungs, collapsed alveoli do not contribute to the ventilation process. Although collapsed alveoli may be properly perfused they contribute to pulmonary shunt because they do not contribute to ventilation. As PEEP is applied, more alveoli are recruited, which means there is more aerated surface area for gas exchange, which causes a decrease in shunt [27,28]. Thus, if it is possible to estimate changes in shunt, then the question is whether the diffusion model can work backwards to predict changes in CO?

If shunt decreases with increased recruitment, then it is possible to estimate shunt changes using the lung mechanics model shown. Because the lung mechanics model has the ability to estimate changes in recruitment [15,16], it is hypothesised here that the increase in recruited volume due to PEEP is directly related to the decrease in shunt. More specifically, changes in recruited volume are assumed equal to reductions in shunt.

If an initial shunt value is known, then to estimate a percentage change in shunt, changes in end expiratory lung volumes (EEV) are examined with reference to the F_h_. With reference to Figure 3 the percentage change in shunt (Δf_s_) is evaluated:

(8) $$\text{Δ}{\text{f}}_{\text{s}}=\frac{\text{E}\text{E}{\text{V}}_{1}-\text{E}\text{E}{\text{V}}_{2}}{{\text{F}}_{\text{h}}-\text{E}\text{E}{\text{V}}_{1}}$$

Although the lung mechanics model cannot predict absolute shunt volumes, it does have the ability to predict changes in shunt volume. Thus, for the first PEEP setting, a shunt value/volume is measured using the diffusion model with a known or estimated CO. Once an initial shunt measurement is obtained, lung mechanics can be used to estimate the shunt at a new level of PEEP by calculating the percentage change in shunt from that initial value.

**Figure 7:**
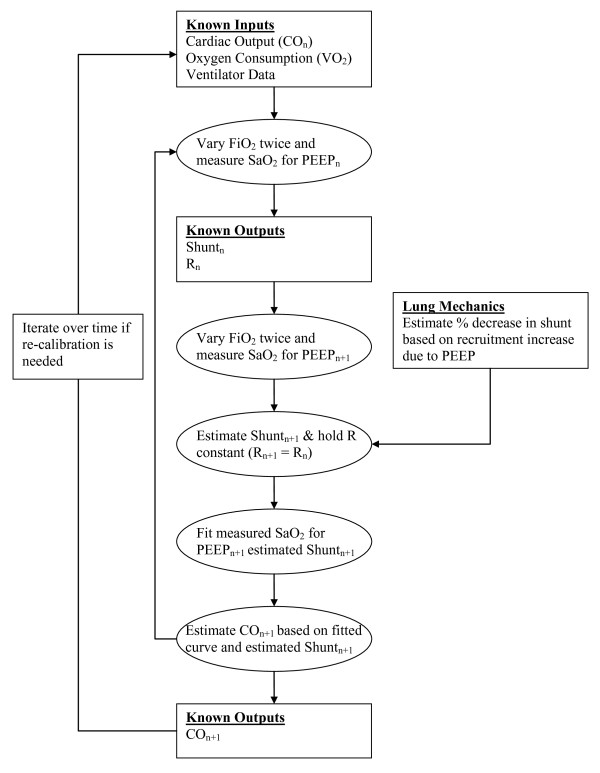
**Flow chart determining the process of estimating cardiac output at a higher PEEP level**.

Given that result, subsequent measurements of F_I_O_2 _and S_a_O_2 _at the new PEEP, and fitting an S_a_O_2 _curve constrained by the new level of shunt means the level of CO can be estimated working backwards to find the CO value that yields this curve. This overall process of evaluating the CO is summarised in Figure 7.

In particular, Figure 7 shows the need for an initial measurement of CO. Once an initial measurement or estimate is obtained, it is then possible to track changes in CO with changes to PEEP. Thus, over a given time period, the changes in CO are calibrated to the initial CO measurement obtained through thermodilution.

## Model Validation and Analysis Results

### Proof of Concept

To test the concept of tracking changes in CO, a proof of concept (POC) model has been evaluated based on a mixture of simulated parameters and clinical data. The data used in the study uses one set of data containing PV loops obtained from Bersten et al [29] and arterial blood gas measurements from Andreassen et al [14]. Although these measurements do not correspond to the same data set, the aim is to evaluate the utility of the POC model prior to further clinical validation.

Three PV loops were obtained from Bersten et al [29] with the associated deflation to EEV using constant tidal volume. The TOP and TCP were modelled using the lung mechanics model [15,16] as shown in Figure 3 and in Table 3. Using Equation (8), it is then possible to evaluate the percentage change in shunt as PEEP increases.

**Table 3:** Measured PV data from Bersten et al fitted with Yuta lung mechanics model

***PEEP *[cm H**_**2**_**O]**	*EEV *[L]	***F***_***H ***_**[L]**	**Δ*f***_***s***_
5	0.4410		
		
7	0.6740	1.98	-15%
		
12	1.1125		-34%

Because there is no initial measurement of shunt at the first PEEP level, it is not possible to estimate the shunt at higher levels of PEEP. The PV data from Bersten et al did not include any blood gas measurements. To simulate the POC model, the blood gas data from Andreassen [14] was used and is shown in Table 4. To fit the S_a_O_2 _curve, two measurements of F_I_O_2 _were required. These initial F_I_O_2 _and S_a_O_2 _measurements at t_1 _and t_2 _were assumed to occur at the initial PEEP setting of 5 cmH_2_O. It was also assumed that the CO at the first PEEP was 5 L/min. These overall values and assumptions, while not from the same data, are clinically realistic. Measuring S_a_O_2 _at two different F_I_O_2 _values for a given PEEP is also readily achieved.

**Table 4:** Measured values of varying FiO2 and SaO2 from Andreassen et al.

	**Time t**_**1**_	**Time t**_**2**_
*F_I_O_2 _*[%]	25	35

*S_a_O_2 _*[%]	90.9	95.1

These values are assumed to occur at the initial PEEP level

**Figure 8:**
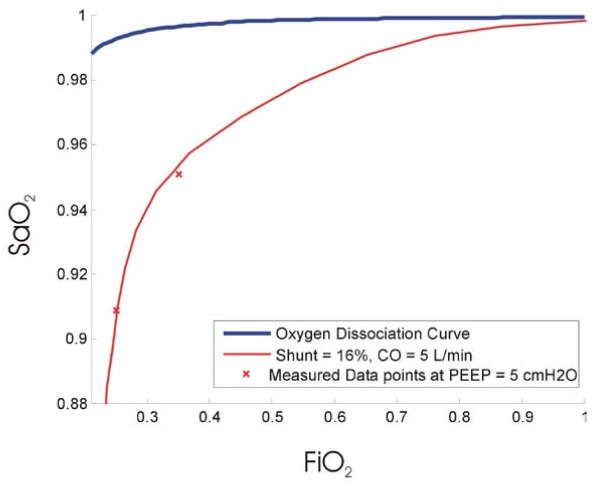
**Best fit oxygen saturation curve assumed to occur at the first PEEP**. Shunt = 16% and diffusion resistance = 45 kPa/L/min with CO = 5 L/min.

Fitting the diffusion model to the data in Table 4 yields a shunt of 16% and a diffusion resistance of 45 kPa/L/min. For the purpose of this POC model, it is assumed that this is the true shunt at the initial PEEP. The raw data points and the best fit S_a_O_2 _curve are shown in Figure 8.

**Figure 9:**
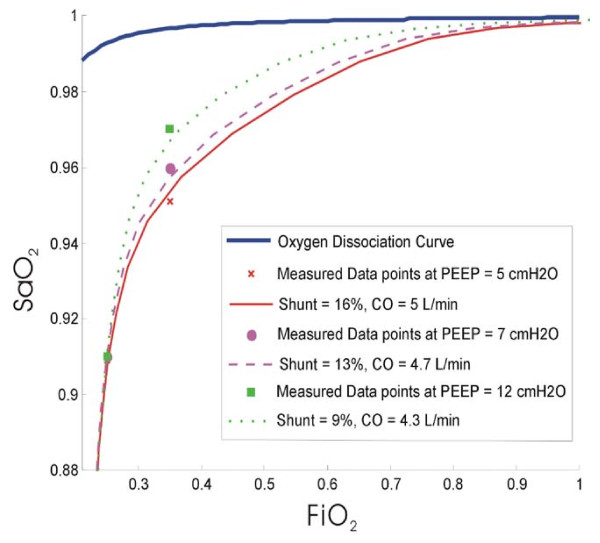
**Improved oxygenation as shown by the increase in oxygen saturation with PEEP application**.

After evaluating the shunt at the initial PEEP level, it is then possible to evaluate the shunt at higher PEEP using the percentage change in shunt from the results in Table 3. For the higher PEEP values, the measured S_a_O_2 _is fit to an oxygen saturation curve by constraining the predicted shunt and thus, evaluating the new CO, which is shown in Figure 9.

The shunt, CO and diffusion resistance for this POC model example are summarised in Table 5 along with the values of F_I_O_2 _and S_a_O_2_.

**Table 5:** Modified shunt and cardiac output as a function of PEEP.

PEEP **[cm H**_**2**_**O]**	***F***_***I***_***O***_***2***_ [%]	***S***_***a***_***O***_***2***_ [%]	Shunt [%]	R [kPa/L/min]	CO [L/min]
5	[25 35]*	[0.909 0.951]*	16*	45	5*

7	[25 35]*	[0.91 0.96]*	13^+^	45	4.7^+^

12	[25 35]*	[0.91 0.97]*	9^+^	45	4.3^+^

* indicates measured or known input values. ^+ ^indicates estimated values

### Optimisation

In the POC model, the cardiac output was shown to drop from 5 to 4.3 L/min as PEEP increased from 5 to 12 cmH_2_O. This drop agrees with current data showing that CO drops with increased PEEP [9,10]. The POC model showed that the application of PEEP improved oxygenation (S_a_O_2_). However, Figure 6 shows that any decrease in CO should reduce oxygenation with all else equal and shunt at a constant value.

**Figure 10:**
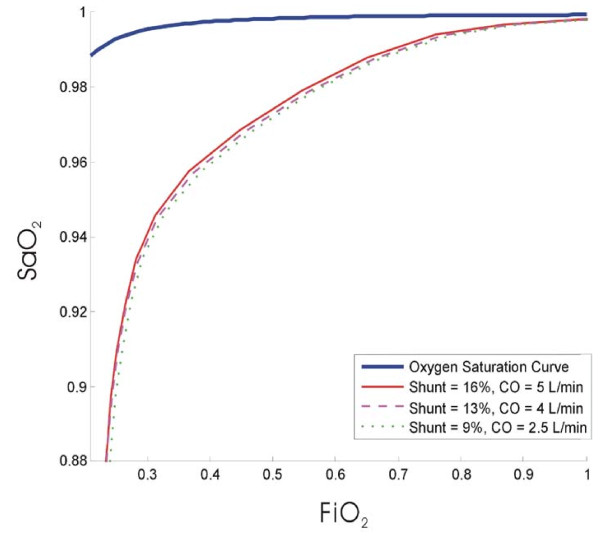
**Decrease/negligible effect on oxygenation due to PEEP indicating that there is a tradeoff between decreased shunt and decreased CO when PEEP is applied**.

The results of the POC model thus confirm the trade off between the amounts of decreased shunt versus the decrease in cardiac output. To test this theory, the CO was set to drop from 5 to 2.5 L/min (corresponding to a drop of 0.5 L/min/cmH_2_O) as PEEP increased, for the same shunt values shown in Table 5. The resulting oxygen saturation curves are shown in Figure 10 and it is evident that if the drop in cardiac output is too high, then the application of PEEP and resulting increased volume for gas exchange does not improve oxygenation, as seen by the minimal gap between curves in Figure 10 versus Figure 9. In addition, S_a_O_2 _curves in Figure 10 actually drop as PEEP is increased. Such a situation clinically would thus require increased F_I_O_2_, with its own risks [30], to improve S_a_O_2_.

Figure 10 shows that if the drop in CO is too high, it can have a detrimental effect on oxygenation even with decreased shunt. The decrease in CO offsets the positive effects of PEEP application and causes decreased oxygenation. More importantly, the ability to capture this effect in this analysis suggests that this model can be used to evaluate oxygenation based on changes in cardiac output due to PEEP.

**Figure 11:**
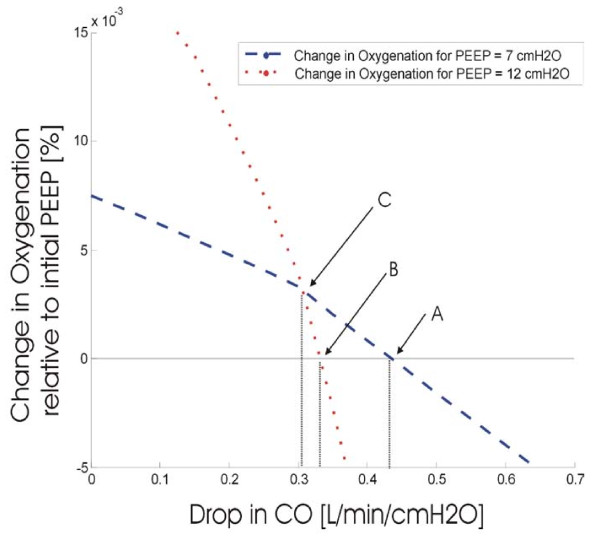
**Effect of drop in cardiac output on oxygenation**.

Figure 11 shows the effect on oxygenation depending on the magnitude of the cardiac output drop as PEEP is applied. The dashed curve represents the oxygenation change when PEEP is 7 cmH_2_O and the shunt is estimated at 13%, while the dotted curve is for PEEP of 12 cmH_2_O and shunt at 9%. The curves give an indication of the maximum allowable drop in CO for a given shunt that will not offset the benefit of increasing PEEP. Three distinct points are shown; A, B and C.

Point A represents the maximum allowable drop in cardiac output (approximately 0.43 L/min/cmH_2_O) at which application of PEEP 7 cmH_2_O will still yield beneficial oxygenation. However, at point A, if the PEEP is then raised to 12 cmH_2_O, the oxygenation is worse off. Thus, point B is the maximum allowable CO drop if a PEEP of 12 cmH_2_O is to be applied (approximately 0.33 L/min/cmH_2_O). Between points B and C, an applied PEEP of 12 cmH_2_O will provide better oxygenation than the initial PEEP, but still not as good as provided by PEEP of 7 cmH_2_O. Only if the drop in CO is less than 0.3 L/min/cmH_2_O (Point C), then a PEEP of 12 cmH_2_O is more beneficial than 7 cmH_2_O.

Thus, based on the drop in cardiac output as a function of PEEP, this model-based approach provides a means of optimising the PEEP setting in ventilation. In the case shown in Figure 11 the ideal PEEP levels are determined by the drop in CO and summarised in Table 6. Hence, during an initial recruitment manoeuvre, if the drop in cardiac output can be estimated, then based on the rate of change of CO due to PEEP, an optimal PEEP can be selected. More importantly, the entire process requires only an initial estimate of CO or a single invasive thermodilution measurement. From that point, it can noninvasively track changes in CO as required, although the need to recalibrate is not yet known and will require clinical verification.

**Table 6:** Optimum level of PEEP depending on rate of cardiac output change

**PEEP [cmH**_**2**_**O]**	Drop in cardiac output [L/min/cmH2O]
5	>0.43

7	>0.30 and <0.43

12	<0.30

### Robustness Testing

In clinical practice, the need to initially measure the CO may not be viable due to the severity of the patient's condition, and an estimate for the CO at the base PEEP may be required. To test the validity of the initial CO estimate, a robustness test was conducted.

**Figure 12:**
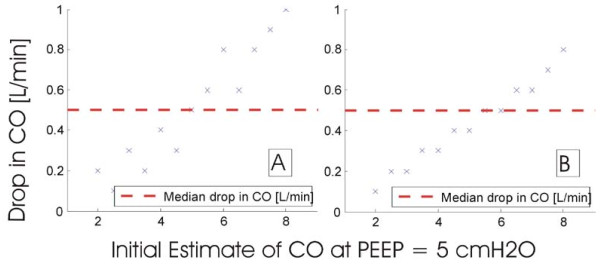
**Scatter plot of drop in CO for different initial estimates of CO relative to median drop**. (A) Drop from changing PEEP from 5 to 7 cmH_2_O, (B) drop from changing PEEP from 7 to 12 cmH_2_O.

**Figure 13:**
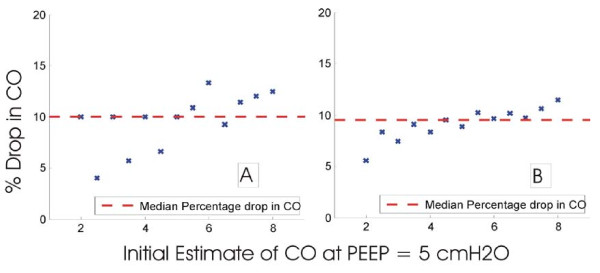
**Scatter plot of percentage drop in CO for different initial estimates of CO relative to median percentage drop**. (A) Drop from 5 to 7 cmH_2_O, (B) drop from 7 to 12 cmH_2_O.

Cardiac output differs between patients and is typically reported to range from 2-8 L/min [24-26]. For the initial PEEP level of 5 cmH_2_O in Figure 9 the CO was varied between 2 and 8 L/min in steps of 0.5 L/min. Using the linear least squares method, a line of best fit was then plotted to fit through the 'measured' data points at a PEEP of 7 and 12 cmH_2_O. The results of the robustness test are shown in Table 7 where the initial estimate of CO at PEEP = 5 cm H_2_O is shown in the grey cells. The drop in CO and percentage drops in CO is shown for the different initial estimates in Figure 12 and 13.

**Table 7:** Initial estimate of CO at PEEP = 5 cmH2O (bold italicised cells).

	PEEP			Drop in CO [L/Min]		Percentage Drop in CO [%]	
	5	7	12	PEEP 5 & 7	PEEP 7 & 12	PEEP 5 & 7	PEEP 7 & 12
**Cardiac** **Output**	***2***	1.8	1.7	0.2	0.1	10	6
	***2.5***	2.4	2.2	0.1	0.2	4	8
	***3***	2.7	2.5	0.3	0.2	10	7
	***3.5***	3.3	3	0.2	0.3	6	9
	***4***	3.6	3.3	0.4	0.3	10	8
	***4.5***	4.2	3.8	0.3	0.4	7	10
	***5***	4.5	4.1	0.5	0.4	10	9
	***5.5***	4.9	4.4	0.6	0.5	11	10
	***6***	5.2	4.7	0.8	0.5	13	10
	***6.5***	5.9	5.3	0.6	0.6	9	10
	***7***	6.2	5.6	0.8	0.6	11	10
	***7.5***	6.6	5.9	0.9	0.7	12	11
	***8***	7	6.2	1	0.8	13	11
	**Median**			*0.5*	*0.4*	*10*	*9.5*
	**Max**			*1.0*	*0.8*	*13.3*	*11.4*
	**Minimum**			*0.1*	*0.1*	*4.0*	*5.6*

Drop in CO and percentage drop in CO shown for the different initial estimates.

Figure 12 shows the effect of different initial estimates of CO on the absolute drop in CO at higher PEEP values. The graph illustrates the absolute drop on CO to be highly dependent on the initial estimate. As the initial estimate of CO becomes higher, the drop in CO also increases, and Figure 12 shows significant differences from the median CO drop.

However, from a percentage drop perspective, Figure 13 indicates that the percentage drop does not vary too much. The percentage drop of CO with the application of PEEP is approximately constant and does not drop by more than 15%. It is also clear that all points are within close proximity to the median. Thus, given an arbitrary initial estimate, it is possible to track percentage changes as a function of PEEP application.

### Sensitivity to Lung Mechanics

The estimation of percentage change in shunt is calculated using Equation (8), and is dependent on the maximum theoretical lung capacity, as determined by the lung mechanics model. However, the theoretical lung capacity is a value derived from the model fit and is not the exact value of true lung capacity. Thus, the estimation of shunt changes according to Equation (8) may not be clinically accurate.

To test how the change in shunt is affected by different values of theoretical lung capacity, a sensitivity test was performed. By varying the theoretical lung capacity by 10%, the effect on the shunt at a PEEP of 7 cmH_2_O and the percentage decrease in shunt between 5 and 7 cmH_2_O was modelled to measure the sensitivity of these two parameters.

**Figure 14:**
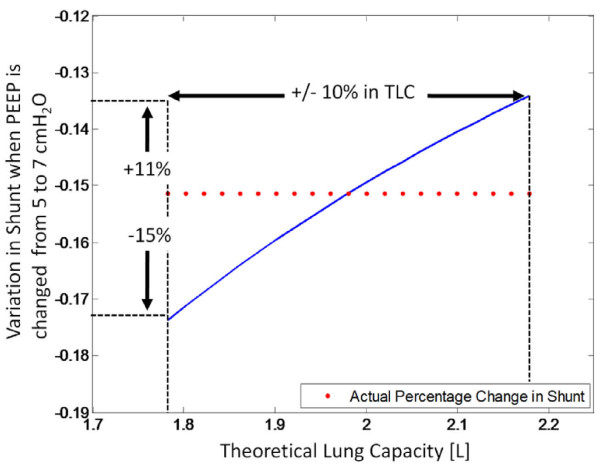
**Effect on the percentage drop in shunt from 5 to 7 cmH_2_O as the theoretical lung capacity is varied by 10%**.

As shown in Figure 14 a 10% change in the theoretical lung capacity causes the percentage drop on shunt between PEEP of 5 and 7 cmH_2_O to be between 10 and 15%. Although this is still within what is clinically tolerable, it indicates that the percentage drop is reasonably sensitive to the lung capacity.

**Figure 15:**
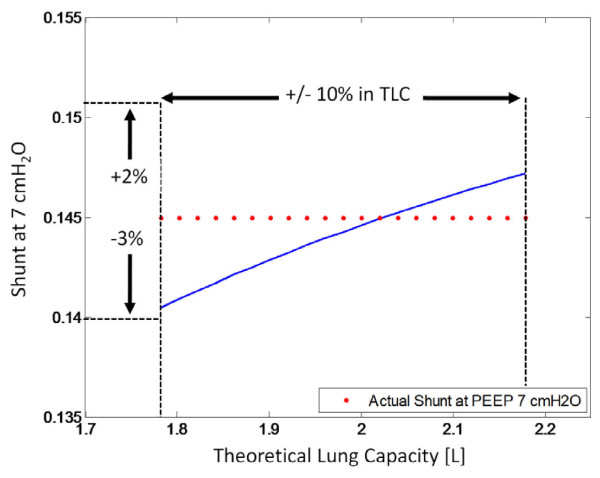
**Effect on shunt at PEEP 7 cmH_2_O as the theoretical lung capacity is varied by 10%**.

However, if the absolute value of shunt is examined, then there is very minimal difference when the lung capacity is varied. Figure 15 shows that for a 10% change in lung capacity, the shunt evaluated at a PEEP of 7 cmH_2_O only varies by a maximum of 3%. This apparent lack of sensitivity illustrates that even though the lung mechanics model does not estimate the true lung capacity, one can be relatively confident that the shunt at higher PEEP levels is reasonably accurate given the initial shunt measurement is known.

## Model Limitations and Discussion

According to the diffusion model, as CO drops, the S_a_O_2 _should also drop given no other parameters change. However, an application of PEEP is generally used to improve oxygenation and cause an increase in S_a_O_2, _even when PEEP causes CO to drop. The POC model highlights the tradeoff that can occur when PEEP increases and CO drops. When PEEP is applied in the POC model, S_a_O_2 _can increase. Although increasing PEEP causes a drop in CO, the S_a_O_2 _can still increase if the beneficial effect of PEEP on oxygenation offsets the detrimental effect of a CO drop on oxygenation.

The model this paper presents has some limitations that must also be discussed. The first major limitation is the lack of complete clinical data in validating this model. Although the aim of this paper was to describe the proof of concept, the combination of two different data sets does not give an accurate representation of true lung mechanics. However, this model will be tested on real clinical haemodynamic and lung mechanics data, which will be obtained in recently approved trials. Equally importantly, as noted, the values obtained are still well within reported ranges lending some added credibility to the analysis presented.

Although the application of PEEP causes a decrease in CO, CO itself is affected by different factors [25]. In reality, it is difficult to know what effect various combinations of ventilation parameters and lung mechanics have on CO without any further clinical data. The work by Crotti et al [1] indicated that CO did change significantly as PEEP increased. However, in that paper, tidal volume was not kept constant across PEEP. The model presented here used PV data with constant tidal volume. Thus, it is reasonable to assume that with all other parameters constant, the sole application of PEEP will lower CO. However, the model is limited as it does not examine the effect on CO when other parameters are varied and more clinical data is required.

The paper uses the diffusion model of Andreassen et al [14]. When evaluating the changes in shunt due to PEEP application, the model assumes that diffusion resistance does not change for the purpose of simplicity. It also considers and treats shunt as being homogeneous and responsive to PEEP, which is not always the case. These assumptions may or may not be accurate and will be tested with further clinical data.

The estimations of shunt changes according to Equation (8) are dependent on the total lung capacity as modelled by the lung mechanics model. In reality, this theoretical lung capacity could take on any value and thus, the estimation of shunt changes may not be entirely accurate. However, the lung mechanics model is currently being validated in clinical trials. If the lung mechanics model accurately predicts recruitment as compared to computed tomography scans, then this assumption of shunt change should be accurate enough.

Finally, the initial measurement of shunt requires a known value of cardiac output. In this study, the initial cardiac output for the PEEP of 5 cmH_2_O was assumed to be 5 L/min. This measurement may require some invasive measurement that may not be ideal for all MV patients. Furthermore, without the use of clinical data, it is difficult to see how frequently the CO needs to be re-calibrated to the initial measurement from thermodilution. However, even if the initial cardiac output is estimated, the changes in CO are still tracked, which is the more important parameter. Thus, the initial measurement of the initial CO can be avoided if the changes in CO is all that is needed, provided that a reasonably accurate estimate is available.

## Conclusions

This article has developed a model of lung mechanics and gas exchange. It has two primary applications. First, it can be used to monitor CO and assess the impact of changes in PEEP on the resulting CO. Hence, it can, secondly, potentially be used to optimise PEEP with respect to gas exchange and oxygenation, as well as its impact on circulation and its management.

More specifically, two models are presented and linked through a hypothesis that a change in shunt can be approximated by a change in lung volumes as PEEP changes. A proof of concept case study based on clinical data is used to show the model's capability and validity. Finally, sensitivity studies are used to illustrate the models potential robustness.

Such linked physiological models offer the opportunity to move beyond simple clinical, model-based decision support to more complex cases including physiological interactions between systems. The results presented show promise and justify further clinical validation in upcoming clinical trials.

## Competing interests

The authors declare that they have no competing interests.

## Authors' contributions

AS, JGC & CH developed the model presented in the paper. GS provided some of the clinical data used in the study. All authors read and approved final manuscript.

## Ackknowledgements

N/A
